# Tarlov Cyst Rupture and Intradural Hemorrhage Mimicking Intraspinal Carcinomatosis

**DOI:** 10.7759/cureus.15423

**Published:** 2021-06-03

**Authors:** Tejaswi Sudhakar, Likowsky L Désir, Jason A Ellis

**Affiliations:** 1 Neurosurgery, Lenox Hill Hospital Northwell Health, New York, USA; 2 Medicine, City University of New York School of Medicine, New York, USA

**Keywords:** perineural cyst, spine hemorrhage, intradural tumor, leptomeningeal carcinoma, tarlov cyst

## Abstract

Lumbosacral Tarlov cysts (TCs) have rarely been seen to rupture. Here, we report an unusual presentation of a ruptured TC with intraspinal hemorrhage mimicking carcinomatosis. Pathological diagnosis was obtained using percutaneous biopsy. A conservative approach was utilized and an excellent outcome was achieved. Thus, in cases such as this, a ruptured hemorrhagic TC should be on the differential diagnosis to drive appropriate clinical management decisions.

## Introduction

Tarlov cysts (TCs), also known as perineural cysts, are spinal intradural cerebrospinal fluid (CSF)-filled sacs that form within the nerve root sleeves. These lesions are often found in the sacral region of the spinal column. Though predominantly asymptomatic, TCs may compress spinal nerve roots and clinically present with low back or leg pain and radiculopathy [1,2]. To our knowledge, hemorrhagic presentation of a ruptured TC mimicking carcinomatosis has not been previously reported. Here, we present such a case and detail our conservative management strategy.

## Case presentation

**Figure 1:**
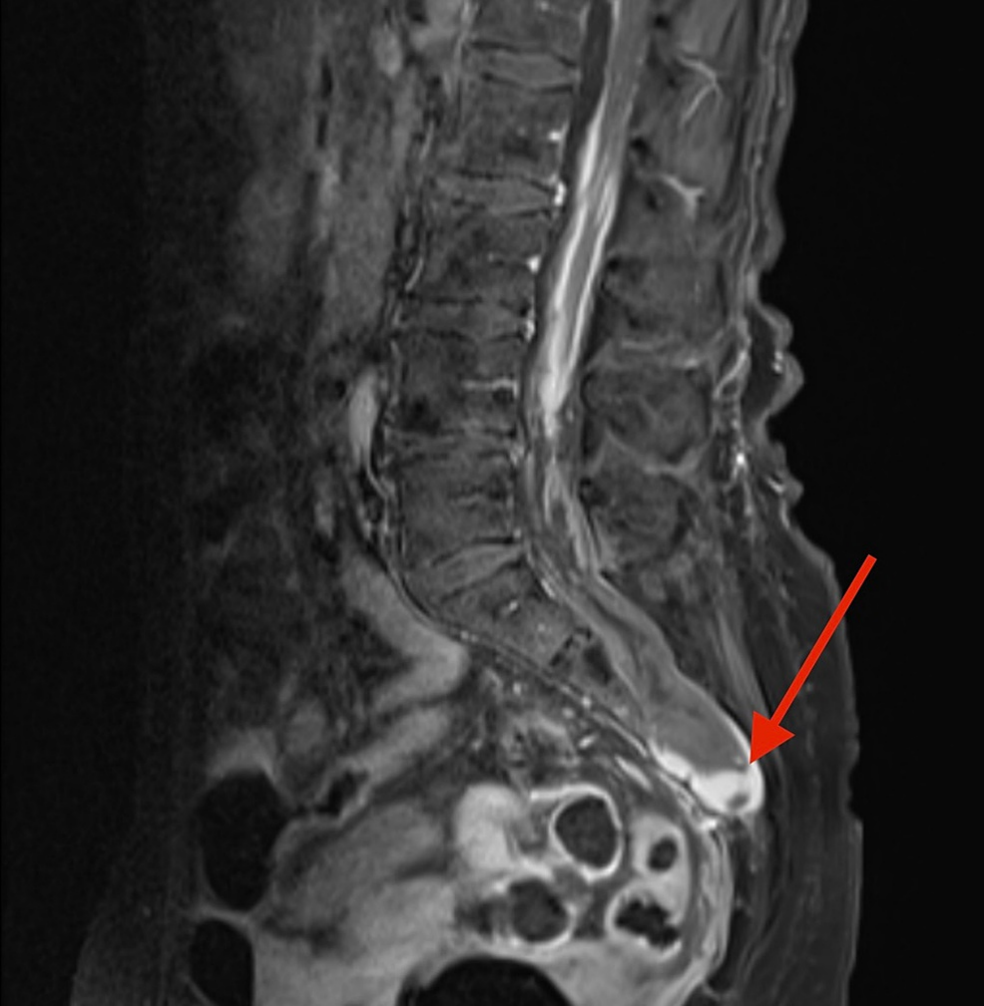
Sagittal T1 -weighted MRI with contrast revealing a high signal intensity 3 cm cystic lesion in the lumbosacral spine at S3-S4. Red arrow points to the lesion.

**Figure 2:**
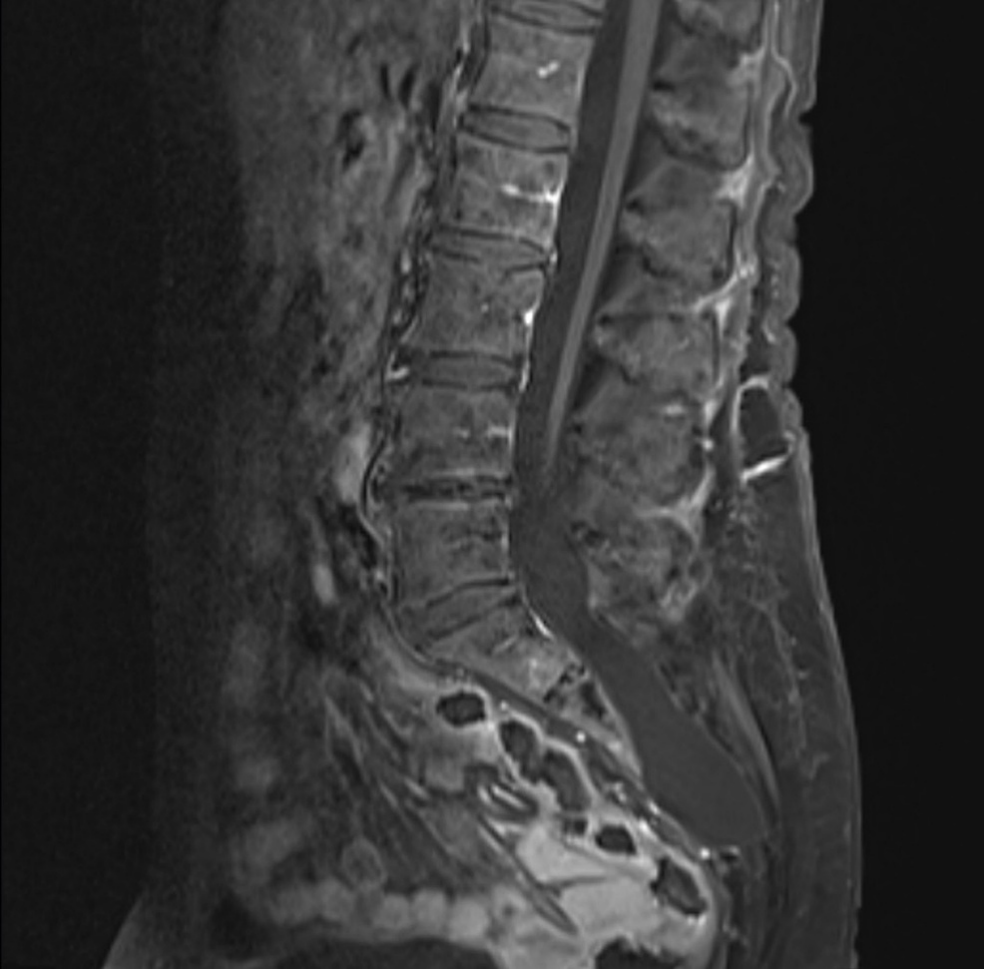
Postoperative six-month follow-up. Sagittal T1-weighted MRI with contrast revealing complete resolution of the intraspinal hemorrhage, with expansion of the sacral spinal canal and cystic dilation.

A 71-year-old woman with no significant past medical history presented with acute-onset headache, severe low back pain, and urinary retention. Physical examination was notable for sacrococcygeal tenderness to palpation and pain-limited antigravity motor strength in the legs. Head imaging was unremarkable. Lumbosacral MRI demonstrated a 3 cm heterogeneously enhancing mass-like lesion at the S3-S4 level. Additionally, diffuse cauda equina nerve root and leptomeningeal enhancement were noted (Figure 1, red arrow). A preliminary diagnosis of leptomeningeal carcinomatosis was rendered based on the imaging profile. A CT-guided needle biopsy of the sacral mass was performed, revealing organized hematoma and no evidence of malignant tissue. With this histological result, a decision was made to pursue conservative management, especially as the patient had progressive clinical improvement after the presentation. The patient was neurologically intact at the six-month follow-up. MRI at that time demonstrated complete resolution of the intraspinal hemorrhage and enhancement. Continued expansion of the sacral spinal canal and cystic dilation of the nerve root sleeves were indicating TC (Figure 2).

## Discussion

TCs, or perineural cysts, are intradural CSF-filled sacs found within the lumbosacral spine [3]. They arise at or just distal to the dorsal root ganglion between the layers of the endo- and perineurium [4-10]. The incidence of TC is estimated to be between 1.5% and 4.6% with a prevalence of approximately 13% [11-13]. Kuhn et al. reported a 13.2% detection frequency of perineural cysts in 1,100 consecutive sacral MRI studies [14]. TCs are reported to be present in 4.0% of people less than 50 years old and in 1.3% of people greater than 50 years old [3]. Although the literature is not definitive, case reports and series suggest a female preponderance [3,7,8,10-13,15-18].

TCs are most commonly asymptomatic and are generally found incidentally during radiographic studies performed for other reasons. Symptomatic TCs have a reported incidence of 1% in the adult population [11,12,19]. Enlarging TCs become symptomatic due to compression of the adjacent bone, perineural vasculature, or nerve fibers [20]. It has been proposed that TCs lacking significant communication with the subarachnoid space are more likely to be asymptomatic [15]. The etiology of TCs remains unclear although one theory proposes that stenosis at the ostium of the nerve root sleeve allows CSF influx with arterial systolic pulsation coupled with restricted outflow [9,11,12,19]. Myelographic demonstration of subarachnoid microcommunication supports a valve mechanism and differentiates TCs from other cystic osteolytic lesions within the lumbosacral space [18]. Surgical intervention is not indicated for the majority of symptomatic and asymptomatic TCs. Alternatively, percutaneous cyst drainage, fibrin glue injection, insertion of cyst-subarachnoid or lumboperitoneal shunts, laser ablation, duraplasty, and microsurgical fenestration have been advocated in select cases [8,14].

Acute symptomatic presentation with cyst rupture and hemorrhage as seen in this case is quite rare. Godel et al. reported a case of a 38-year-old woman who developed acute radiculopathy in such a case [20]. Yates et al. reported the case of a 61-year-old man who developed symptomatic nerve root compression associated with cyst rupture [5]. Both cases resolved spontaneously without the need for surgical intervention. As seen in our case, complicated TCs can mimic intraspinal neoplasia in both clinical and radiographic findings. One report by Attiah et al. presented the case of a giant sacral schwannoma mimicking a TC [16].

## Conclusions

TC rupture resulting in symptomatic intraspinal hemorrhage is rare. The possibility of symptomatic TCs merits a high degree of suspicion, especially if radiographic findings are suggestive of a neoplastic etiology. Conservative management is indicated in such a setting where TC is suspected, including cautious monitoring for clinical deterioration.
